# *Euryops pectinatus* L. Flower Extract Inhibits P-glycoprotein and Reverses Multi-Drug Resistance in Cancer Cells: A Mechanistic Study

**DOI:** 10.3390/molecules25030647

**Published:** 2020-02-03

**Authors:** Wafaa M. Elkady, Iriny M. Ayoub, Yousra Abdel-Mottaleb, Mohamed F. ElShafie, Michael Wink

**Affiliations:** 1 Department of Pharmacognosy, Faculty of Pharmaceutical Sciences and Pharmaceutical Industries, Future University in Egypt, New Cairo 11835, Egypt; welkady@fue.edu.eg; 2 Department of Pharmacognosy, Faculty of Pharmacy, Ain Shams University, Cairo 11566, Egypt; 3 Department of Pharmacology, Toxicology and Biochemistry, Faculty of Pharmaceutical Sciences and Pharmaceutical Industries, Future University in Egypt, New Cairo 11835, Egypt; yabdelmottaleb@gmail.com; 4 Department of Pharmacology and Toxicology, Faculty of Pharmacy, Al-Azhar University, Cairo 11884, Egypt; mohamed.elshfie@su.edu.eg; 5 Department of Pharmacology and Toxicology, Faculty of Pharmacy, Sinai University, East Kantara—New City, El Ismailia 41636, Egypt; 6 Institute of Pharmacy and Molecular Biotechnology, Heidelberg University, INF 364, D-69120 Heidelberg, Germany

**Keywords:** *Euryops pectinatus*, LC/MS, cytotoxicity, P-glycoprotein, multidrug resistance, bromodomains, molecular docking

## Abstract

*Euryops pectinatus* is a South African ornamental plant belonging to family Asteraceae. The present work evaluates the cytotoxic activity and phytochemical profile of the flower extract. Metabolite profiling was performed using HPLC-PDA-ESI-MS/MS. Total phenolics and flavonoids content were assessed. Cytotoxicity was evaluated against 6 different cancer cell lines using MTT assay. The possible underlying mechanism was proposed. We analyzed whether the extract could overcome the resistance of multidrug-resistant cancer cells for doxorubicin. The effect of combination of *E. pectinatus* with doxorubicin was also studied. Additionally, the potential inhibitory activity of the identified phytochemicals to PB1 protein was analyzed using in silico molecular docking. Twenty-five compounds were tentatively identified. Total phenolic and flavonoid contents represented 49.41 ± 0.66 and 23.37 ± 0.23 µg/mg dried flower extract, respectively. The extract showed selective cytotoxicity against Caco2 cells but its main effect goes beyond mere cytotoxicity. It showed strong inhibition of P-glycoprotein, which helps to overcome multidrug resistance to classical chemotherapeutic agents. In silico molecular docking showed that dicaffeoyl quinic acid, kaempferol-*O*-rutinoside, rutin, and isorhamnetin-*O*-rutinoside exhibited the most potent inhibitory activity to PB1 involved in tumor progression. *Euryops pectinatus* flower heads could have promising selective cytotoxicity alone or in combination with other chemotherapeutic agents to counteract multidrug resistance.

## 1. Introduction

The development of drug resistance during cancer chemotherapy limits the survival rate of cancer patients [1]. Multidrug resistance (MDR) renders cancer cells immune to standard treatments with various anti-cancer drugs and is a major limitation of cancer therapy [2]. To overcome MDR in cancer treatment, many strategies have been proposed, including chemotherapy with a combination of anti-tumor drugs and an inhibitor of P-glycoprotein (P-gp) and other ABC transporters. P-gp belongs to the first member of the ATP-binding cassette (ABC) superfamily, which is encoded by the MDR1 gene [2], and is widely expressed in epithelial cells of normal tissues that are involved in drug disposition, including the liver, intestine, and kidney. It has been reported that P-gp and other ABC transporter can actively bind and pump out drugs against the concentration gradient by an ATP-powered membrane drug efflux pump; therefore, anticancer drugs are prevented from accumulation inside the cells to trigger cytotoxic effects. The intracellular concentration of some commonly used anticancer drugs is reduced by P-gp and other ABC transporter, including vincristine, etoposide, and doxorubicin [3]. The first generation of P-gp inhibitors included verapamil, trifluoperazine, cyclosporine-A, quinidine, reserpine, vincristine, yohimbine, tamoxifen, and toremifene, which are all competitive substrates of P-glycoprotein [3]. Natural compounds isolated from medicinal plants are promising leads in the development of novel chemotherapeutic agents [4]. 

*Euryops pectinatus* (L.) Cass (Asteraceae) is native to South Africa and is widely used as an ornamental plant. It is commonly known as woolly resin bush, golden daisy bush or golden Euryops [5]. The genus name is from the Greek eurys, meaning 'large' and ops, meaning 'eye', referring to its showy flowers. The species name *pectinatus*, is Latin meaning 'pectinate' with narrow divisions like a comb, denoting the divided leaves [5]. Flavonoids, furoeremophilanes, eremophilanolides, and secofuroeremophilanes were identified from the genus *Euryops* [6]. Essential oil of *E. arabicus* leaves was rich in sesquiterpene hydrocarbons and oxygenated sesquiterpenes [7]. The hexane extract of *E. pectinatus* flower-heads was dominated by *n*-pentacosane in addition to phytosterols in the unsaponifiable fraction, whereas, linoleinic acid was the major constituent in the saponifiable fraction [8]. Another species, *E. arabicus* displayed in vivo antioxidant and protective effects against hepatorenal toxicity induced by paracetamol in rats [6]. *E. pectinatus* leaf essential oil displayed potent in vitro cytotoxic activity against Ehrlich ascites carcinoma cells (EAC) and human brain cells (U251) [9]. 

Only few phytochemical and pharmacological reports have been published from *E. pectinatus*. In this context, this study aimed to evaluate the metabolic profile and anti-proliferative activity of *E. pectinatus* flower heads. The possible underlying mechanism of action was also evaluated. Moreover, the inhibitory activity of the identified phytochemicals to PB1 protein involved in tumour progression was investigated using in silico molecular docking. 

## 2. Results and Discussion 

### 2.1. Phytochemical Analysis

#### 2.1.1. Quantitation of Total Phenolic and Flavonoid Content 

Quantitative analysis of the EP-MF revealed the presence of considerable amount of both phenolic and flavonoid constituents; 49.41 ± 0.66 µg/mg and 23.37 ± 0.23 µg/mg dried flower extract respectively. 

#### 2.1.2. Metabolites Profiling of *E. pectinatus* Flower Heads Methanolic Fraction

**Figure 1:**
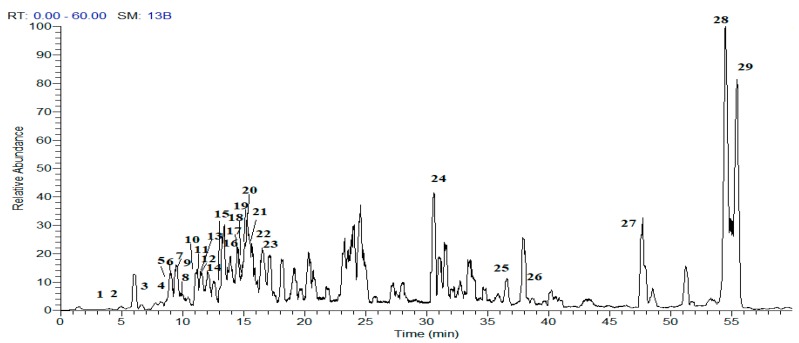
HPLC-ESI-MS base peak chromatogram of *E. pectinatus* flower heads in the negative ion mode. Peak numbers correspond to compounds listed in Table 1.

A comprehensive metabolic profiling of the EP-MF was performed using HPLC-PDA-ESI-MS/MS. Chromatograms were acquired in both negative and positive ion modes (Figure 1). A total of 29 peaks were detected, of which 25 metabolites were tentatively identified based on their UV spectra as well as MS/MS data given by the mass of the molecular ion peak and their fragments, considering the neutral mass loss and known fragmentation patterns as well as comparison with the available literature. A list of identified peaks together with their chromatographic and spectroscopic data is presented in Table 1. The identified compounds belonged to various classes, including organic acids, phenolic acids, flavonoids and fatty acids. 

##### Phenolic Acids and Their Derivatives

In the present work, fourteen phenolic acids were characterized from EP-MF, mainly, caffeic acid, quinic acid, protocatechuic acid and sinapic acid derivatives. Peak 3 exhibited a deprotonated molecular ion at *m*/*z* 153, and a product ion at *m*/*z* 109 via the loss of CO_2_. Thus, peak 3 could be annotated as protocatechuic acid [10]. Chlorogenic acid could be detected at 8.35 min (Peak 4), where it showed [M − H]^−^ at *m*/*z* 353 and MS*^2^* ions at *m*/*z* 191 (quinic acid) and *m*/*z* 179 (caffeic acid).

Peaks 10 and 12 showed quasi molecular ion peaks at *m*/*z* 367 and exhibited a product ion at *m/z* 191 indicating a quinic acid moiety, following the loss of a ferulic acid moiety. Thus, peaks 10 and 12 could be identified as feruloyl quinic acid isomers [11,12]. Peak 11 was identified as caffeic acid, where it showed a molecular ion peak at *m/z* 179 with a product ion at *m*/*z* 135 owing to the loss of CO_2_ [12,13]. Meanwhile, caffeic acid-*O*-hexoside (peak 5) exhibited a parent ion at *m*/*z* 341 and a fragment ion at *m*/*z* 179 for caffeic acid corresponding the loss of a hexose moiety (−162 amu) [13]. Peaks 7 and 8 exhibited deprotonated molecular ions at *m*/*z* 353 and were assigned as caffeoyl quinic acid isomers. Both compounds displayed similar fragmentation pattern showing product ions in the MS/MS spectrum at *m*/*z* 191 corresponding to quinic acid moiety and a fragment ion at *m*/*z* 179 suggesting a caffeic acid moiety. Peak 13 showed a parent ion at *m*/*z* 223 and MS*^2^* fragments at *m*/*z* 179 resulting from the loss of a CO_2_ fragment thus suggesting a carboxylic acid, followed by a subsequent loss of a methyl group at *m*/*z* 165. Therefore, peak 13 was tentatively identified as sinapic acid [14,15].

##### Flavonol Glycosides

Quercetin, kaempferol, isorhamnetin and syringetin glycosides were the major flavonol glycosides identified in *E. pectinatus* flower heads; quercetin-*O*-glycosides such as quercetin-3-*O*-hexoside and quercetin-3-*O*-rutinoside (rutin) showed characteristic product ion at *m*/*z* 301, corresponding to quercetin aglycone [16]. The nature of the sugars could be revealed by elimination of the sugar residue, that is, 162 amu (hexose; glucose or galactose) and 146 amu (rhamnose) [17]. A kaempferol *O*-glycoside namely, kaempferol-*O*-rutinoside (18), showed a deprotonated molecular ion at *m*/*z* 593 and a product ion at *m/z* 285, corresponding to kaempferol aglycone following the loss of 308 amu (a rutinose) [16]. Additionally, peaks 22 and 23 were tentatively identified as isorhamnetin glycosides; namely, isorhamnetin-*O*-rutinoside and isorhamnetin-*O*-hexoside exhibiting molecular ion peaks at *m*/*z* 623 and 477, respectively. Both exhibited a characteristic product ion at *m*/*z* 315, corresponding to isorhamnetin aglycone, and a loss of 308 (rutinose) and 162 (hexose), respectively [16]. Syringetin-*O*-hexoside (Peak 14) followed the same fragmentation manner, exhibiting a molecular ion peak at *m*/*z* 507 and a characteristic fragment ion at *m*/*z* 345 corresponding to syringetin aglycone and the loss of 162 amu of a hexose moiety. The respective molecular weights were recorded in Table 1 and the fragmentation pattern is illustrated in Figures S1–S12 (Supplementary Materials).

### 2.2. Cytotoxicity of E. pectinatus Using MTT Assay

Cytotoxicity of the extract was evaluated on 6 different cancer cell lines in comparison to two standard drugs: doxorubicin and 5-fluorouracil (Table 2). In general, EP-MF shows moderate cytotoxicity. Results showed that EP-MF exhibited higher cytotoxicity against Caco-2 (IC_50_ = 17.04 μg/mL) compared to 5-fluorouracil (IC_50_ = 20.22 μg/mL). 

#### 2.2.1. IC_50_ Values and Relative Resistance of Doxorubicin and EP-MF in Wild-type and Multidrug-resistant MCF-7/Dox, CEM/ADR5000 Leukemia Cells

As expected, the IC_50_ values of doxorubicin and EP-MF in the MDR cell lines MCF7/Dox and CEM/ADR5000 (Table 3A/B) were higher than in the sensitive parent cell lines (Table 3A/B).

#### 2.2.2. Effects of Combinations of EP-MF and Doxorubicin 

The IC_50_ values of doxorubicin alone and in combination with EP-MF in MDR CEM/ADR5000 and Caco-2 cells were calculated and represented in Table 4. The combination of EP-MF (5 μg/mL) with DOX has increased the potency by almost 50% in MCF/Dox cells and CEM/ADR500 cells, and by 37% in Caco2 cells, approximately. 

#### 2.2.3. Effects of the Extract on P-glycoprotein Activity in CEM/ADR5000 Cells and in Caco-2 Cells

To further investigate the effects shown in Table 3 and Table 4, the inhibition of P-pg by verapamil as a standard drug and EP-MF were assessed in the calcein AM assay (Table 5). The extract had a similar inhibitory activity as verapamil. With regard to the presence of quercetin and rutin in the extract (Table 1), one can speculate about these promising effects. Earlier studies have shown similar effects regarding inhibition of P-glycoprotein by quercitin and rutin, which are considered potential chemosensitizing agents to overcome MDR in cancer. Indeed, there exist a long line of P-glycoprotein inhibitors however, their use is limited by their toxicity and their wide drug interactions. On the other hand, up to 10 µM of quercitin and rutin are well tolerated by patients presenting an excellent potential to overcome MDR in cancer [21]. 

### 2.3. Molecular Docking 

Bromodomains have attracted serious interest over the past few years as promising new epigenetic targets for diverse human diseases as cancer. PB1 protein is considered a critical factor for tumor progression [22]. The inhibitory activity of the identified metabolites from our extract to the PB1 protein was evaluated using in silico molecular docking. The crystal structure of luteolin extracted from PB-1 was superimposed to the docked pose and agreed well with the conformation obtained, leading to an RMSD value between the calculated pose and the crystal structure of 1.9445.

**Figure 2:**
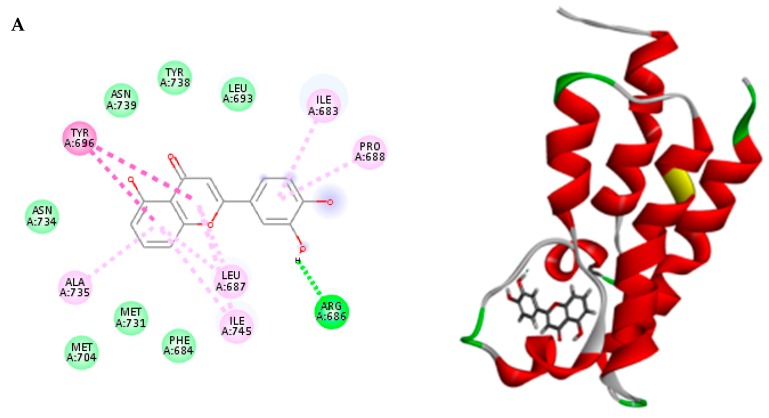

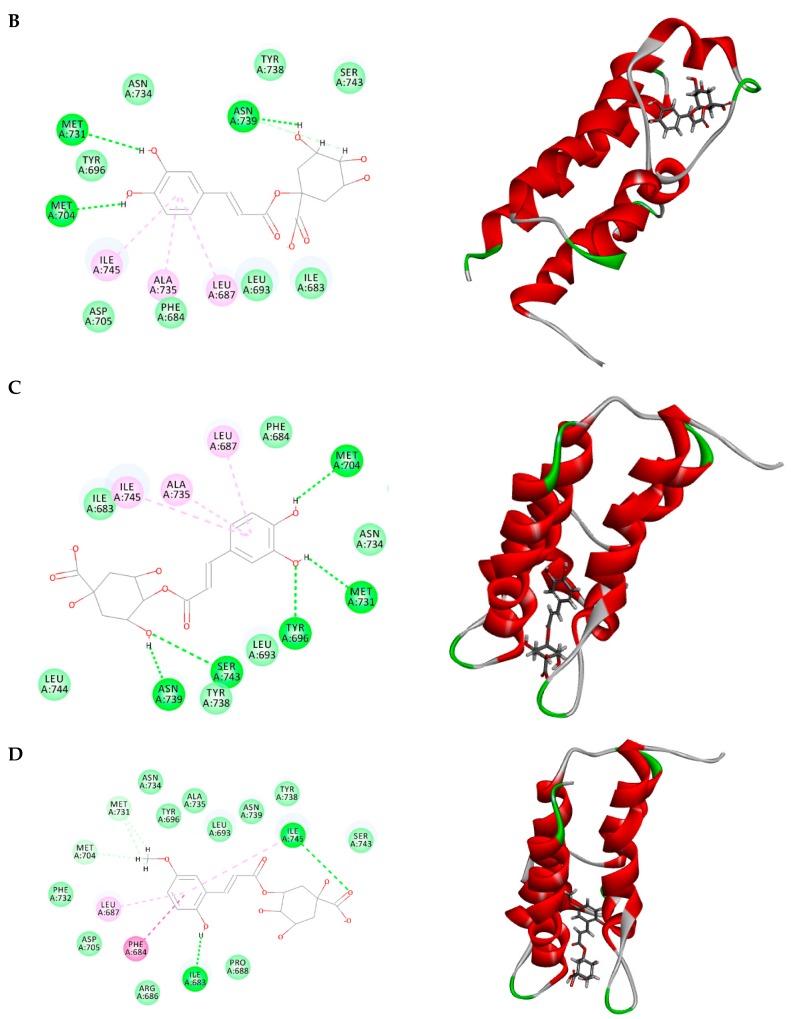

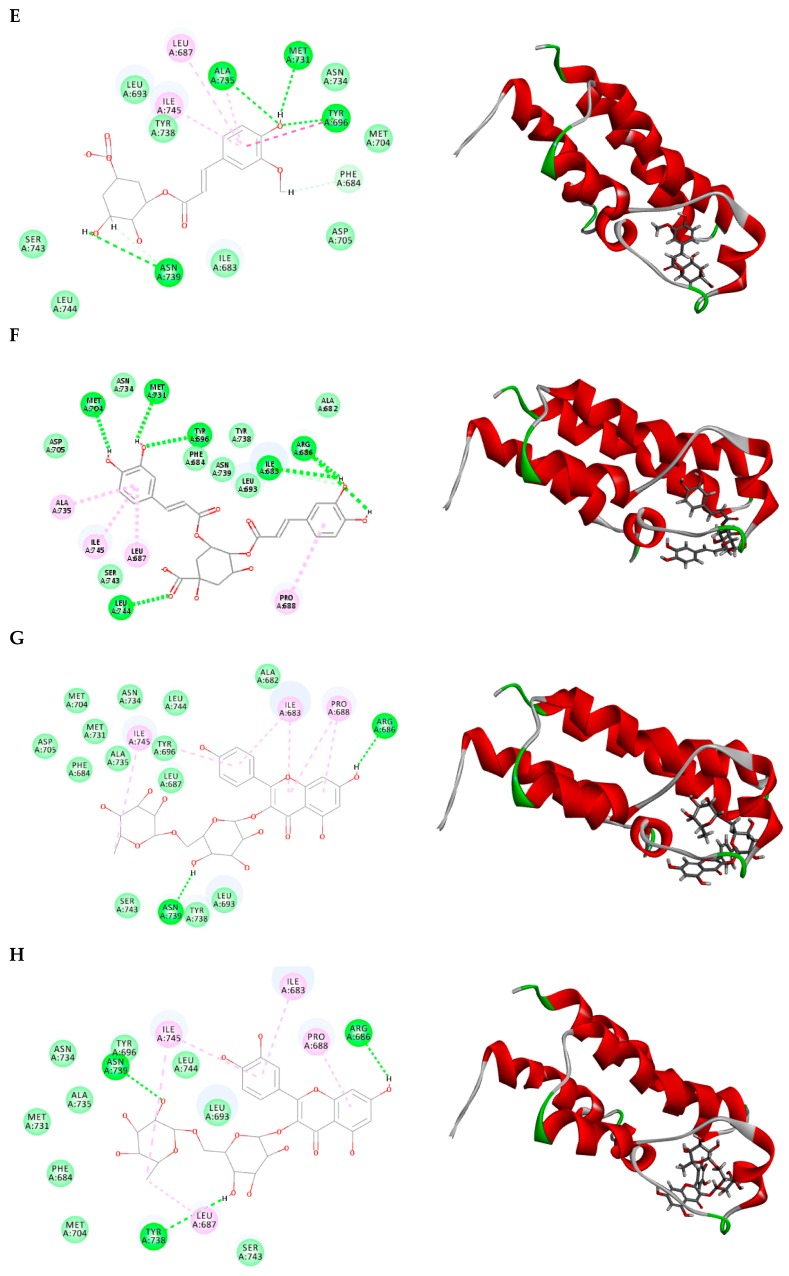

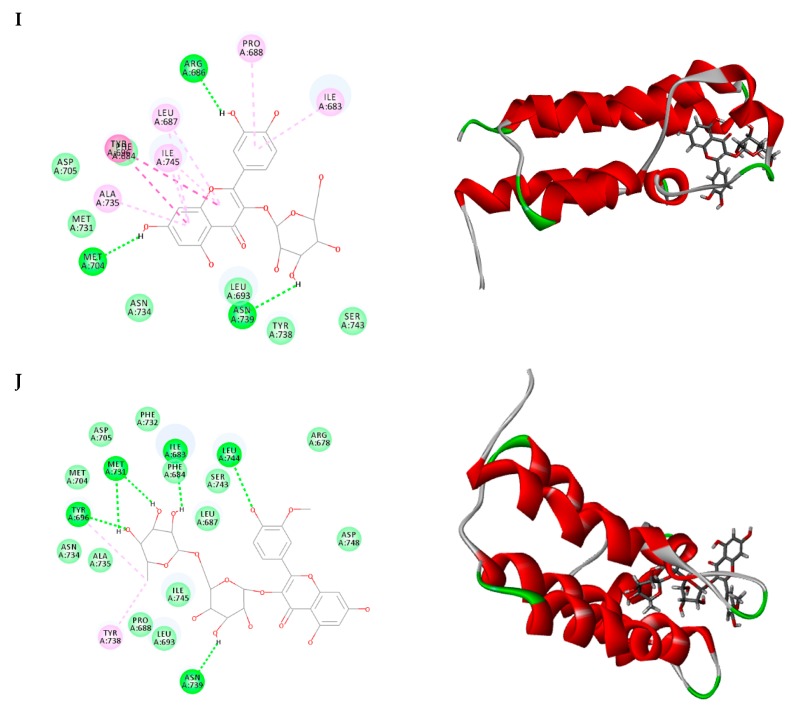
2D and 3D ligand−enzyme interactions of high score compounds: (**A**) Luteolin (LU2), the co-crystallized inhibitor; (**B**) 1-*O*-Caffeoyl quinic acid (**7**); (**C**) 4-*O*-Caffeoyl quinic acid (**8**); (**D**) 3-Feruloyl quinic acid (**10**); (**E**) 5-Feruloyl quinic acid (**12**); (**F**) 3,4-Dicaffeoyl quinic acid (17); (**G**) Kaempferol-*O*-rutinoside (**18**); (**H**) Rutin (**20**); (**I**) Quercetin-*O*-hexoside (**21**); (**J**) Isorhamnetin-*O*-rutinoside (**22**); with PB1 (PDB ID: 5II2). Residues are annotated with three-letter amino acid code and their position. Polar residues are coloured green; hydrophobic residues are coloured purple, Hydrogen-bonding interactions are represented with a green dashed line between the receptor and the ligand while π–alkyl interactions are represented with a purple dashed line.

The ability of the tested phytochemicals to interact with the key amino acids through H-bonding and π–π stacking with different amino acids in the binding site of PB1 receptor rationalizes their efficiency in cytotoxic activity as indicated by their docking pattern and docking score with PB1 receptor protein compared to that of luteolin, the co-crystallized ligand (ca. −35.38 Kcal/mol). Dicaffeoyl quinic acid isomers (**17** and **19**), quercetin-*O*-hexoside (**21**), kaempferol-*O*-rutinoside (**18**), rutin (**20**), isorhamnetin-*O*-rutinoside (**22**), feruloyl quinic acid isomers (**10** and **12**), caffeoyl quinic acid isomers (**7** and **8**) showed the most potent inhibitory activities compared to luteolin as represented by their favorable binding exhibiting high free binding energies to Pb1 active site. The binding energy and pattern for each compound is represented in (Table 6 and Figure 2). Accordingly, it can be concluded that the identified phytochemicals can serve as potential inhibitors for tumor progression.

## 3. Materials and Methods

### 3.1. Chemicals and Reagents

Folin Ciocalteu reagent was obtained from Sigma-Aldrich (St Louis, MO, USA). Reference standards of gallic acid and quercetin were obtained from Sigma-Aldrich (St Louis, MO, USA). Aluminium chloride, was supplied by Al-Nasr Co., Egypt. MTT [3-(4,5-dimethylthiazol-2-yl)-2,5-diphenyltetrazolium bromide) which was purchased from Sigma-Aldrich (Munich, Germany). All other reagents and media were purchased from Invitrogen (Karlsruhe, Germany). Solvents were purchased from Sigma-Aldrich (Munich, Germany).

### 3.2. Plant Material 

*E. pectinatus* flower heads were collected in January 2017, from a public garden in Cairo, Egypt. The plant material was authenticated by Dr. Mohamed El-Gibaly, Professor of Taxonomy and Consultant for Central Administration of Plantation and Environment, Cairo, Egypt. A voucher specimen (PHG-EP-116) has been deposited in the Pharmacognosy Department, Faculty of Pharmacy, Future University in Egypt, New Cairo, Egypt. 

### 3.3. Preparation of Plant Extract

Air-dried flower heads (480 g) were coarsely powdered and exhaustively extracted with 70% aqueous methanol (4 L × 6). The residue left after evaporation of the solvent (90.36 g) was defatted with dichloromethane under reflux (1 L × 5, 50 °C) to yield 3.20 g of the dichloromethane fraction and 85.63 g of the defatted methanol fraction (EP-MF) which was kept in a tight container at 4 °C for further analysis. 

### 3.4. Phytochemical Analysis

#### 3.4.1. Quantitation of Total Flavonoids 

A spectrophotometric method was conducted using AlCl_3_ complex method [23] Quercetin was used as a reference standard. The assay was performed in triplicate. The total flavonoid content was determined from the calibration curve and expressed as µg quercetin equivalent (QE)/mg dried flower extract.

#### 3.4.2. Quantitation of Total Phenolic Compounds

A spectrophotometric method was adopted using the Folin Ciocalteu colourimetric method [24]. Gallic acid was used as a reference standard. The assay was performed in triplicate. The total phenolic content was calculated as µg gallic acid equivalent (GAE)/mg dried flower extract.

#### 3.4.3. HPLC-PDA-ESI-MS/MS Analysis

HPLC-PDA-ESI-MS/MS analysis was performed on a Finnigan LCQ-Duo ion trap mass spectrometer with an ESI source (ThermoQuest) coupled to a Finnigan Surveyor HPLC system (MS pump plus, autosampler, and PDA detector plus) with a EC 150/3 Nucleodur 100-3, reversed phase C18 column (Macherey-Nagel). A gradient of water and acetonitrile (ACN) with 0.1% formic acid (ESI−) and without (ESI+) was applied from 2 to 100% ACN in 60 min at 30 °C. The flow rate was 0.5 mL/min. The injection volume was 20 µL. All samples were measured in the positive and negative ion modes. The MS was operated with a capillary voltage of 10 V, source temperature of 240 °C, and high purity nitrogen as a sheath and auxiliary gas at a flow rate of 80 and 40 (arbitrary units), respectively. The ions were detected in a mass range of 50–2000 *m*/*z*. Collision energy of 35 eV was used in MS/MS for fragmentation. Data acquisitions and analyses were executed by XcaliburTM 2.0.7 software (Thermo Scientific). 

### 3.5. Biological Activity

#### 3.5.1. Cytotoxicity and Cell Proliferation Assays

##### Cell Culture

Six different human tumor cell lines were used including liver (HepG2), breast (MCF-7), lung (A549), colon (Caco2 and HCT-116) as well as acute lymphocytic leukemia (CCRF-CEM, CEM/ADR5000. All cell lines were obtained from American type cell culture collection (ATCC, Manassas, VA, USA) except Human colon adenocarcinoma; Caco-2 (wild type) cells were a gift from Prof. Dr. Gert Fricker, Institute of Pharmacy and Molecular Biotechnology, University of Heidelberg, Heidelberg, Germany. The cells were originally obtained from a 72-year old Caucasian suffering from colon adenocarcinoma (according to the American Type Culture Collection, Rockville, MD, USA). Additionally, Human leukaemia cell lines CCRF-CEM and CEM/ADR5000 cells were obtained from Dr. Axel Sauerbrey, Department of Pediatrics, University of Jena, Jena, Germany. Cells were originally obtained from a 4-year Caucasian female suffering from acute lymphoblastic leukemia.

##### Assessment of Cytotoxicity Using MTT Assay

EP-MF anti-proliferative activity was investigated using MTT assay [25]. Cell lines were suspended in RPMI 1640 medium in Corning^®^ 96-well tissue culture plates, and then incubated for 24 h. The tested extract was then added into 96-well plates (six replicates) to achieve eight concentrations for each group. Six vehicle controls with media were run for each 96 well plate as a control. After incubating for 48 h, the number of viable cells were determined using MTT assay. Briefly, the media were removed and replaced with 100 µL of fresh culture RPMI 1640 medium then 10 µL of 12 mM MTT stock solution (5 mg of MTT in 1 mL of PBS) were added to each well and cells were allowed to metabolize the dye into a colored insoluble formazan crystal for 2 h. The remaining MTT solution was discarded from the wells and the formazan crystals produced by viable cells were dissolved in 200 μL/well acidified isopropanol for 30 min, then, were covered with aluminum foil and shaken continuously using a MaxQ 2000 plate shaker (Thermo Fisher Scientific Inc, Kalamazoo, MI, USA) at room temperature. Absorbance was measured at 570 nm using a microplate reader (SunRise, TECAN, Inc, Morrisville, NC, USA).The cell viability was expressed as percentage of the control. The concentration that induces 50% of maximum inhibition of cell proliferation (IC_50_) was determined using Graph Pad Prism version 5 (San Diego, CA, USA).

##### Assessment of IC_50_ and Relative Resistance Values of Doxorubicin and *E. pectinatus*

This study was designed to evaluate the IC_50_ and relative resistance of multidrug-resistant MCF-7/Dox, CEM/ADR5000 leukemia and Caco-2 monolayer cells for EP-MF and doxorubicin. MCF-7/Dox and CEM/ADR5000 cells (overexpressing P-gp) were seeded at a density of 5 × 10^4^ cells per well into 96-well plates, whereas, Caco-2 cells were seeded at a density of 2 × 10^4^ cells per well. IC_50_ was determined 72 h post-incubation using MTT assay [25]. Each concentration was processed six times, and the corresponding IC_50_ value was calculated from a sample size of 3 runs. 

##### Effects of *E. pectinatus* on Doxorubicin Cytotoxicity

MCF-7/Dox, CEM/ADR5000 cells were seeded at a density of 5 × 10^4^ cells per well into 96-well plates and initially incubated with non-toxic doses of EP-MF (5 μg/mL) for 6 h, followed by serial dilutions of doxorubicin (0.1–50 µM). Caco-2 cells were seeded at a density of 2 × 10^4^ cells per well and initially incubated with non-toxic doses of EP-MF (5 μg/mL) for 6 h, followed thereafter by serial dilutions of doxorubicin (0.01–20 µM). The cell viability was determined 72 h post-incubation using MTT assay. 

#### 3.5.2. Effects of *E. pectinatus* on P-glycoprotein

To unravel a potential action of EP-MF on P-gp, the following assays were performed using fluorescent P-gp substrates.

##### Assessment of P-glycoprotein Activity via Calcein–AM Assay Using Flow Cytometric Technique

For flow cytometry-based Calcein-AM assay, the cells (MCF-7/Dox, CEM/ADR5000) at a density of 2.5 × 10^7^ cells per well were short-term incubated with non-toxic serial dilutions of EP-MF (0.5–10 µg/mL). Calcein was added to a final concentration of 1 µM for 30 min at 37 °C. The intracellular fluorescence was measured using a fluorescence-activated cell sorting system (FACS). Verapamil was used as a positive control. 

##### Calcein–AM Assay Using a Fluoroskan Ascent Plate Reader

Caco-2 monolayers were incubated with non-toxic concentrations of EP-MF (0.1–10 µg/mL) for 2 h at 37 °C. Subsequently, Calcein-AM was added to a final concentration of 1 μM for 30 min at 37°C. The fluorescence was measured using a Fluoroskan Ascent^®^ plate reader (Microplate reader (SunRise, TECAN, Inc., USA). The fluorescence was detected at λ (excitation) = 485 nm and λ (emission) = 520 nm. Each concentration of the tested extract was measured. The intracellular fluorescence was obtained by subtracting the background fluorescence of control wells and Calcein uptake was expressed as % of control. Verapamil served as a positive control.

### 3.6. Molecular Docking 

The probable molecular binding mode between the identified compounds and fifth bromodomain of human polybromo-1 (PB1) was evaluated using the CDOCKER algorithm in Discovery Studio 4.5 (Accelrys Software, Inc., San Diego, CA, USA). Bromodomains (BRDs) are epigenetic interaction domains currently recognized as emerging drug targets for the development of anticancer agents [26]. The crystal structure of BRDs (PDB ID: 5II2) was retrieved from the Protein Data Bank (http://www.rcsb.org/pdb/). Water molecules in the protein were removed, and the protein was refined. The co-crystallized ligand 5II2 was used as the positive control ligand, and the binding site was defined based on the binding of the co-crystallized inhibitor and PB1. Prior to docking, the co-crystallized ligand was removed; then, the prepared ligands were docked into the protein binding site implementing rule-based and pH-based ionization methods, using appropriate parameters. The interaction energy was calculated to analyze the interaction between the ligand and the receptor. For each ligand, the top 10 ligand-binding poses were ranked according to their CDOCKER energies, and the predicted binding interactions were analyzed, from which the best ligand-binding poses were chosen. Validation of CDOCKER as a docking algorithm for PB1 was achieved by calculating the root-mean-square deviations (RMSD) after redocking the co-crystallized ligand to the protein structure using the algorithm as previously described [27]. To evaluate the possible interactions between the isolated compounds and PB1 proposed binding sites and molecular docking studies were performed using the CDOCKER algorithm within Discovery Studio 4.5.

## 4. Conclusions

In this study, a flower extract from *E. pectinatus* exhibited moderate cytotoxic effect against six types of cancer cell lines. The extract could inhibit P-gp comparable to verapamil and could resensitize MDR cells when applied in combination with doxorubicin. This effect can be attributed to the flavonoid content of *E. pectinatus*. Furthermore, the identified phytochemicals showed promising inhibitory activity to PB1 protein evaluated using in silico molecular docking, thus, can serve as potential inhibitors for tumor progression. The use of *E. pectinatus* extract alone or in combination with conventional chemotherapy might be an interesting candidate as an adjuvant drug in cancer therapy, provided more studies are conducted to determine the in vivo efficacy. 

## Figures and Tables

**Table 1:** Metabolites assigned in *E. pectinatus* flower heads using HPLC-PDA-ESI-MS/MS in negative ion mode.

Compound		*Rt* (min)	[M − H]^−^	Fragment Ions	UV–vis (λ_max_ nm)	Class	References
**1**	Malic acid	4.05	133	115, 99	261	Organic acid	
**2**	Cinnamic acid	4.15	147	129, 115	261	Phenolic acid	
**3**	Protocatechuic acid	6.61	153	109	257, 290	Phenolic acid	[10]
**4**	Chlorogenic acid	8.35	353	191, 179	319, 379	Phenolic acid	[11]
**5**	Caffeic acid-*O*-hexoside	8.99	341	179, 135	319	Phenolic glycoside	[12,13]
**6**	Quinic acid	9.14	191	173, 127, 111	216, 323	Organic acid	[10]
**7**	Caffeoyl quinic acid	9.48	353	191, 179	324	Phenolic acid	[17]
**8**	Caffeoyl quinic acid (isomer)	9.90	353	191, 179	324	Phenolic acid	[17]
**9**	Caffeoyl quinic acid dimer	9.57	707	645, 514, 456, 353	290, 322	Phenolic acid	
**10**	Feruloyl quinic acid	11.04	367	191, 173	295, 322	Phenolic acid	[11,12]
**11**	Caffeic acid	11.18	179	135	297, 324	Phenolic acid	[12,13]
**12**	Feruloyl quinic acid isomer	11.47	367	191, 173	295, 322	Phenolic acid	[11,12]
**13**	Sinapic acid	11.59	223	205, 179, 163	296, 341	Phenolic acid	[14,15]
**14**	Syringetin-3-*O*-hexoside	12.15	507	345	289, 325	Flavonol glycoside	[18]
**15**	Dicaffeoyl quinic acid hexoside	13.09	677	516	289, 324	Phenolic glycoside	
**16**	Caffeoyl malonylhexoside	13.79	427	409, 265, 179. 135	291, 324	Phenolic glycoside	
**17**	Dicaffeoyl quinic acid	14.56	515	515, 353, 191, 179	253, 300, 333	Phenolic acid	[13]
**18**	Kaempferol-*O*-rutinoside	14.84	593	431, 285	253, 332	Flavonol glycoside	[16]
**19**	Dicaffeoyl quinic acid	14.94	515	353, 191, 179	253, 300, 333	Phenolic acid	
**20**	Quercetin-3-*O*-rutinoside (Rutin)	15.23	609	343, 301	254,348	Flavonol glycoside	[19]
**21**	Quercetin-3-*O*-hexoside	15.43	463	301	255, 351	Flavonol glycoside	[19,20]
**22**	Isorhamnetin-*O*-rutinoside	16.52	623	315, 300, 255	253, 292, 338	Flavonol glycoside	[20]
**23**	Isorhamnetin-3-*O*-hexoside	17.20	477	315, 314	288, 336	Flavonol glycoside	[16]
**24**	Unidentified	30.66	344	258, 226	282	Unknown	
**25**	Unidentified	36.57	507	407, 283, 231, 153	279	Unknown	
**26**	Hydroxy-octadecadienoic acid	38.73	295	295, 277	282	Fatty acid	[17]
**27**	Hydroxyhexadecanoic acid	47.66	271	271, 254, 242, 226	277	Fatty acid	[17]
**28**	Unidentified	54.56	817	577, 559, 538. 443, 317, 285	279	Unknown	
**29**	Unidentified	55.42	815	785, 733, 606, 560, 538, 483, 415, 278, 235	275	Unknown	

**Table 2:** Cytotoxicity of *E. pectinatus* using MTT assay.

Drug IC_50_ (μg/mL)	MCF-7	HepG2	A549	Caco-2	HCT-116	CCRF-CEM
Doxorubicin	0.44	0.977	5.842	8.508	6.87	0.033
5-Fluorouracil	1.71	4.12	10.32	20.22	18.33	1.22
*E. pectinatus*-MF	23.32	16.15	52.12	17.04	31.55	28.76

**Table 3:** IC_50_ and relative resistance values of doxorubicin and EP-MF (A) in MCF-7 and multidrug-resistant MCF-7/Dox. (B) in CCRF-CEM and multidrug-resistant CEM/ADR5000 cells.

***A.*** **Drug**	**MCF-7** **IC_50 (_** **µg** **/mL)** **Mean ± SE**	**MCF-7/Dox** **IC_50_ (** **µg** **/mL)** **Mean ± SE**	**Relative Resistance**
Doxorubicin	0.44 ± 0.032	16.82 * ± 1.12	38.23
*E. pectinatus-*MF	23.32 ^a^ ± 1.52	65.92 ^a,^* ± 3.78	2.83
***B.*** **Drug**	**CCRF-CEM** **IC_50_** **µg** **/mL** **Mean ± SE**	**CEM/ADR5000** **IC_50_** **µg** **/mL** **Mean ± SE**	**Relative Resistance**
Doxorubicin	0.033 ± 0.006	4.57 * ± 0.41	138.48
*E. pectinatus-*MF	28.76 ^a^ ± 1.86	97.42 ^a,^* ± 4.36	3.39

Data are presented as means ± SE, *n* = 4. Cells were incubated for 72 h. MTT assay was used for assessment of cytotoxicity. Relative resistance is calculated as the degree of drug resistance (IC_50_ of MCF-7/Dox divided by IC_50_ of MCF-7 and IC_50_ of CEM/ADR5000 divided by IC_50_ of CCRF-CEM)). ^a^: Significantly different from doxorubicin at *p* < 0.05 using one-way analysis of variance (ANOVA) followed by Dunnett as post-hoc test. *: Significantly different from MCF-7 at *p* < 0.05 using unpaired Student’s *t*-test in Table 3A and *: Significantly different from CCRF-CEM leukemia cells at *p* < 0.05 using unpaired Student's t-test in Table 3B.

**Table 4:** Effects of EP-MF on doxorubicin cytotoxicity in MCF-7/Dox, CEM/ADR5000, and Caco-2 cells.

	**MCF-7/Dox** **IC_50_ (μg/mL)** **Mean ± SE**	**CEM/ADR5000** **IC_50_ (μg/mL)** **Mean ± SE**	**Caco-2 cells** **IC_50 (_μg/mL)** **Mean ± SE**
Doxorubicin	16.82 ± 1.12	4.57 ± 0.41	8.50 ± 0.65
DOX + *E. pectinatus*-MF	7.64 ^a^ ± 0.54	2.18 ^a^ ± 0.22	5.28 ^a^ ± 0.73

Data are presented as means ± SE, *n* = 4. Cells were incubated with *E. pectinatus* for 6 h, then followed by doxorubicin thereafter for 72 h. ^a^: Significantly different from doxorubicin at *p* < 0.05 using one-way analysis of variance (ANOVA) followed by Dunnett as post-hoc test.

**Table 5:** Effects of *E. pectinatus-*MF on intracellular calcein fluorescence in CEM/ADR5000 cells and Caco2 cells.

**Compound**	**CEM/ADR5000** **Intracellular Calcein Fluorescence (% Control) Mean ± SE**			
	**Concentration**			
	**0.5 μg/mL**	**2.5 μg/mL**	**5 μg/mL**	**10 μg/mL**
**Control**	100 ± 0	100 ± 0	100 ± 0	100 ± 0
**Verapamil**	100.54 ± 4.24	167.63 ^a^ ± 9.41	268.24 ^a^ ± 11.42	324.4 ^a^ ± 18.71
***E. pectinatus*-MF**	102.01 ± 6.82	162.54 ^a^ ± 10.82	284.63 ^a^ ± 21.42	296.9 ^a^ ± 12.05
**Compound**	***Caco-2*** **Intracellular Calcein fluorescence (% Control) Mean ± SE**			
	**Concentration**			
	**0.5 μg/mL**	**2.5 μg/mL**	**5 μg/mL**	**10 μg/mL**
**Control**	100 ± 0	100 ± 0	100 ± 0	100 ± 0
**Verapamil**	103.71 ± 8.67	165.63 ^a^ ± 13.78	220.25 ^a^ ± 11.12	304.13 ^a^ ± 12.55
***E. pectinatus*-MF**	103.67 ± 7.01	173.92 ^a^ ± 13.86	221.74 ^a^ ± 20.17	288.22 ^a^ ± 16.64

Data are presented as means ± SE, *n* =6. CEM/ADR5000 cells were incubated for 1.5 h. Calcein–AM assay was achieved using Flowcytometry. ^a^: Significantly different from control at *p* < 0.05 using one-way analysis of variance (ANOVA) followed by Dunnett as post-hoc test. Caco-2 cells were incubated for 2 h. Calcein–AM assay was achieved using a Fluoroskan Ascent plate reader. ^a^: significantly different from control at *p* < 0.05 using one way analysis of variance (ANOVA) followed by Dunnett as post-hoc test.

**Table 6:** Free binding energies (ΔG) of the identified compounds within the PB1 active site calculated in kcal/mol using Discovery Studio 4.5 adopting both rule-based and pH-based ionization techniques.

Compound	Binding Energy ΔG (Kcal/mol)	
	Rule-based	pH-based
Luteolin (LU2)	−35.58	−35.58
Malic acid (**1**)	−22.80	−22.80
Cinnamic acid (**2**)	−22.16	−22.16
Protocatechuic acid (**3**)	−22.52	−27.03
Chlorogenic acid (**4**)	−34.63	−37.35
Quinic acid (**6**)	−23.99	−23.99
1*-O-*Caffeoyl quinic acid (**7** or **8**)	−41.14	−36.80
4*-O-*Caffeoyl quinic acid (**7** or **8**)	−35.94	−40.48
3-Feruloyl quinic acid (**10** or **12**)	−44.29	−38.26
Caffeic acid (**11**)	−28.83	−26.33
5-Feruloyl quinic acid ((**10** or **12**)	−44.70	−37.76
Sinapic acid (**13**)	−33.58	−28.39
Dicaffeoyl quinic acid (**17** or **19**)	−50.49	−49.04
Kaempferol-*O*-rutinoside (**18**)	−43.79	−53.74
Rutin (**20**)	−41.21	−50.12
Quercetin-*O*-hexoside (**21**)	−48.53	−49.53
Isorhamnetin-*O*-rutinoside (**22**)	−36.76	−52.02

## Supplementary Materials

The following figures are available online: ESI-MS/MS spectra of selected compounds identified in *E. pectinatus* flower extract.

## Abbreviations

HPLC-PDA-ESI-MS	High performance liquid chromatography-photodiode array-electrospray ionization-mass spectrometry
*E. pectinatus*	*Euryops pectinatus*
EP-MF	*Euryops pectinatus* defatted methanol fraction
HepG2	liver hepatocellular carcinoma cell line
Caco-2	colon carcinoma cell line
MCF7	breast cancer cell line
A549	lung cancer cell line
HCT 116	colon cancer cell line
CCRF-CEM	acute lymphocytic leukemia cell line
MTT	3-(4,5-Dimethylthiazol-2-yl)-2,5-diphenyltetrazolium bromide
MDR	multidrug resistance
P-gp	P-glycoprotein
ABC	ATP-binding cassette
PB1	human polybromo-1
BRDs	Bromodomains
