# Supplementary material for: Euryops pectinatus L. Flower Extract Inhibits P-glycoprotein and Reverses Multi-Drug Resistance in Cancer Cells: A Mechanistic Study
Source: Molecules. 2020 Feb 3;25(3):647. doi: 10.3390/molecules25030647 (PMC7038149; doi:10.3390/molecules25030647)
Supplement: Supplementary file 1 [file molecules-25-00647-s001.pdf]

Supplementary Material

Euryops pectinatus L. flower extract inhibits P-glycoprotein and reverses multi-drug resistance in cancer cells: A mechanistic study

Wafaa M. Elkady [1], Iriny M. Ayoub [2,*], Yousra Abdel-Mottaleb [3], Mohamed F. ElShafie [4,5] and Michael Wink [6,*]

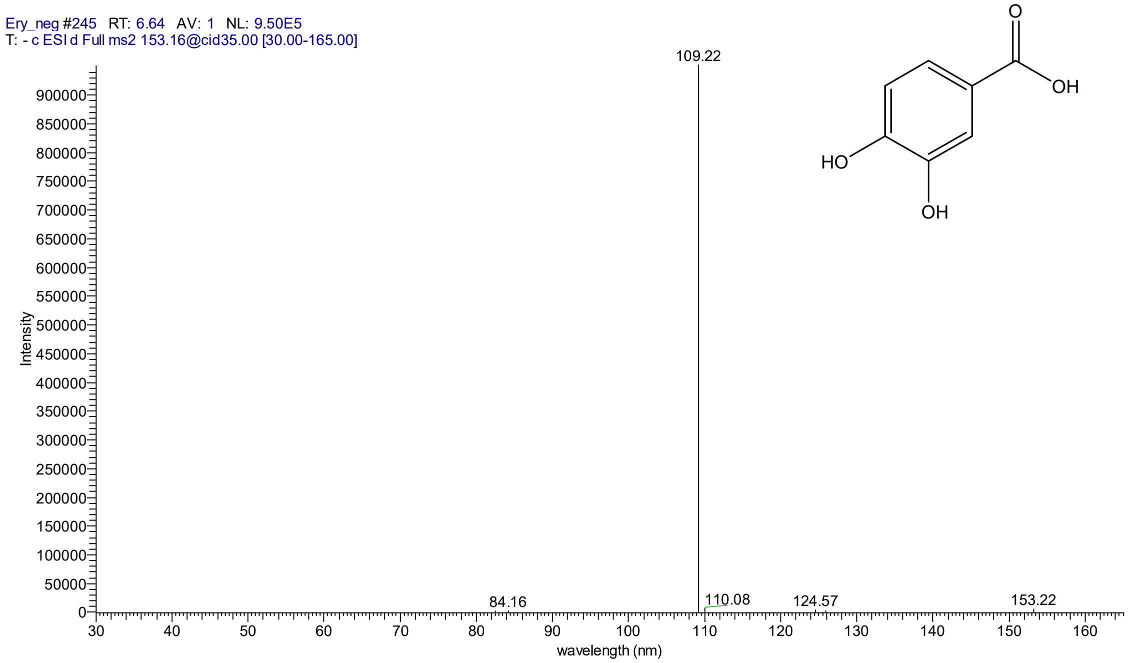

Figure S1. ESI-MS/MS spectrum of protocatechuic acid (**3**) $[M-H]^-$ at *m/z* 153

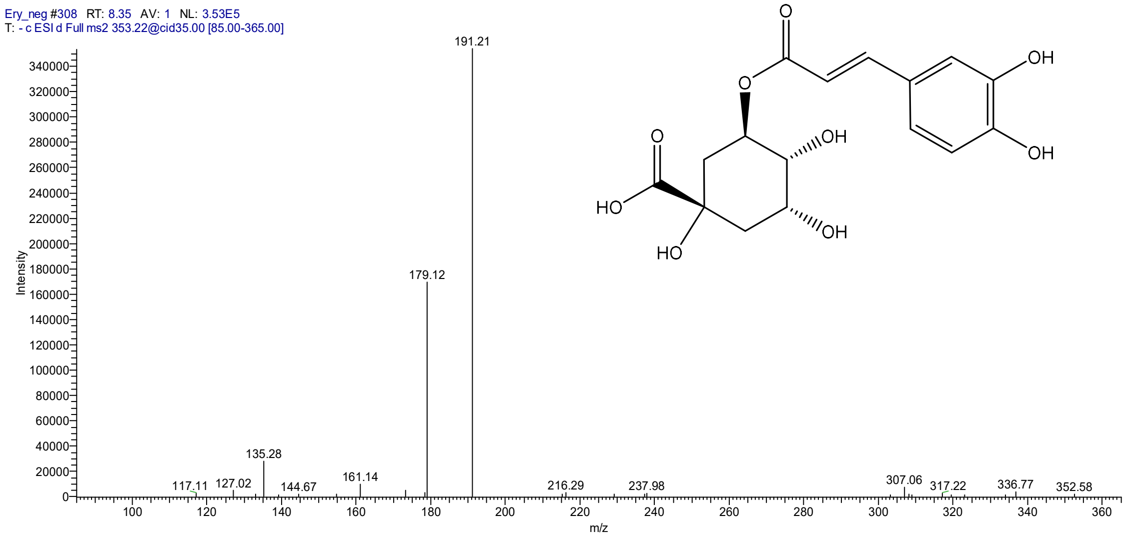

Figure S2. ESI-MS/MS spectrum of chlorogenic acid (**4**) $[M-H]^-$ at *m/z* 353

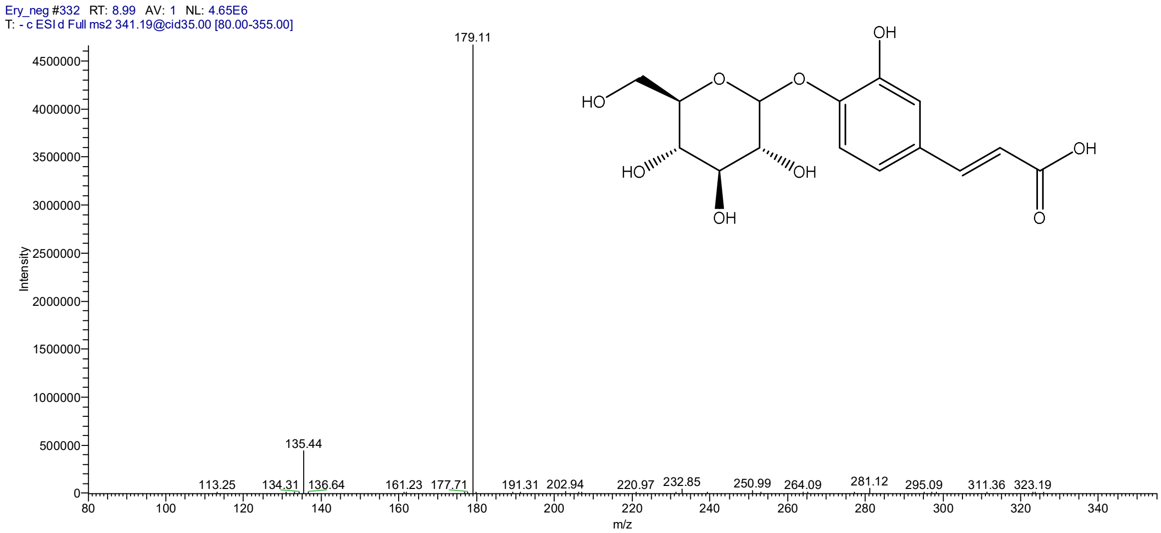

Figure S3. ESI-MS/MS spectrum of caffeic acid-*O*-hexoside (**5**) $[M-H]^-$ at *m/z* 341.19

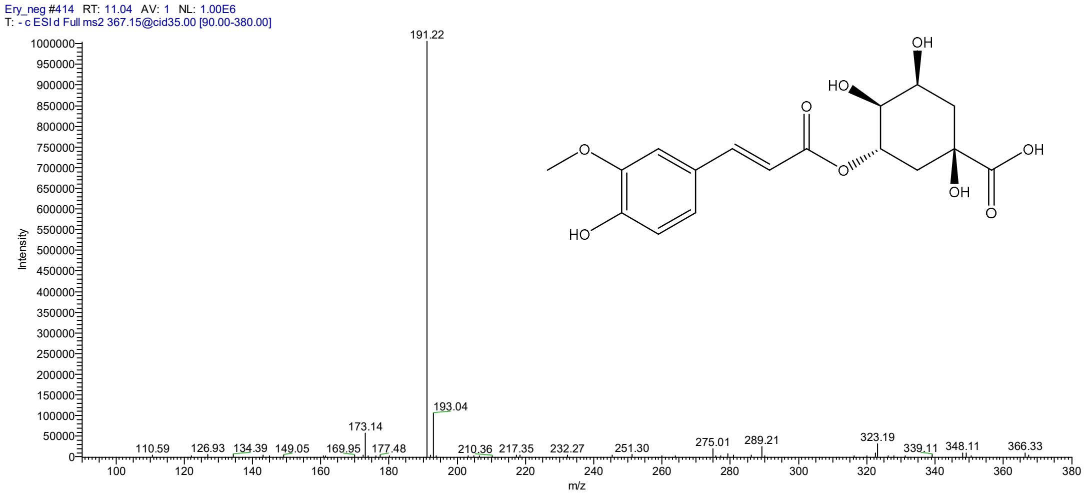

Figure S4. ESI-MS/MS spectrum of feruloyl quinic acid (**10**) $[M-H]^-$ at *m/z* 367.15

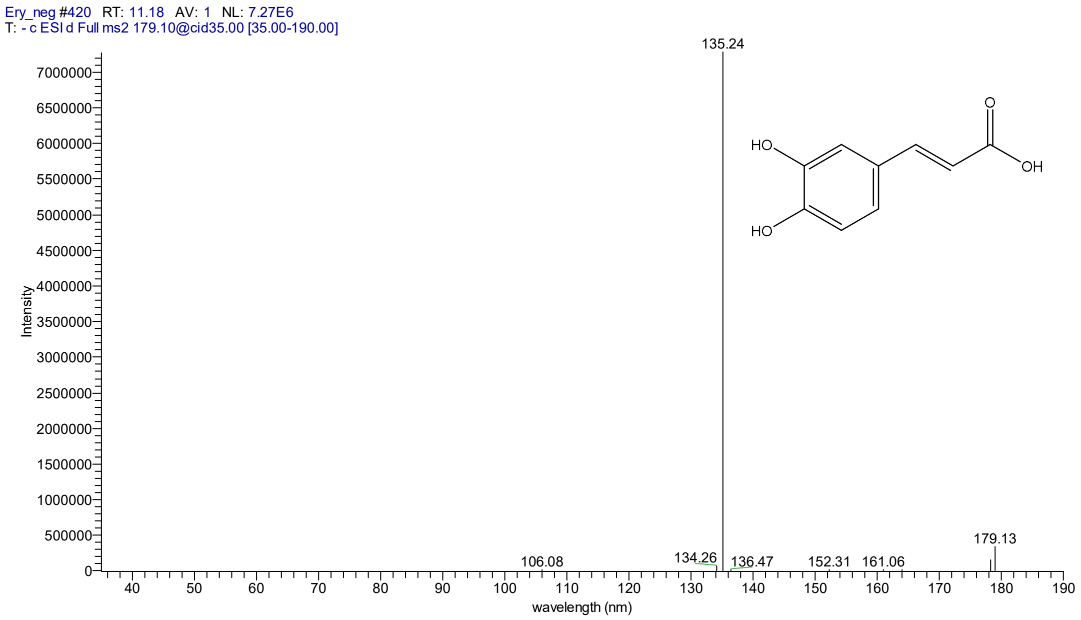

Figure S5. ESI-MS/MS spectrum of caffeic acid (**11**) [M-H]$^{-}$ at *m/z* 179

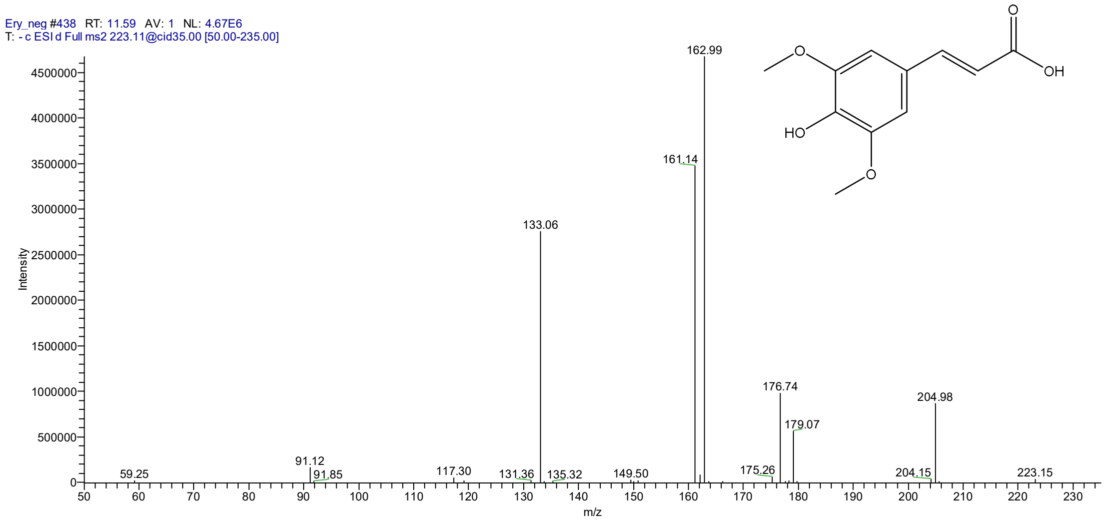

Figure S6. ESI-MS/MS spectrum of sinapic acid (**13**) [M-H]$^{-}$ at *m/z* 223

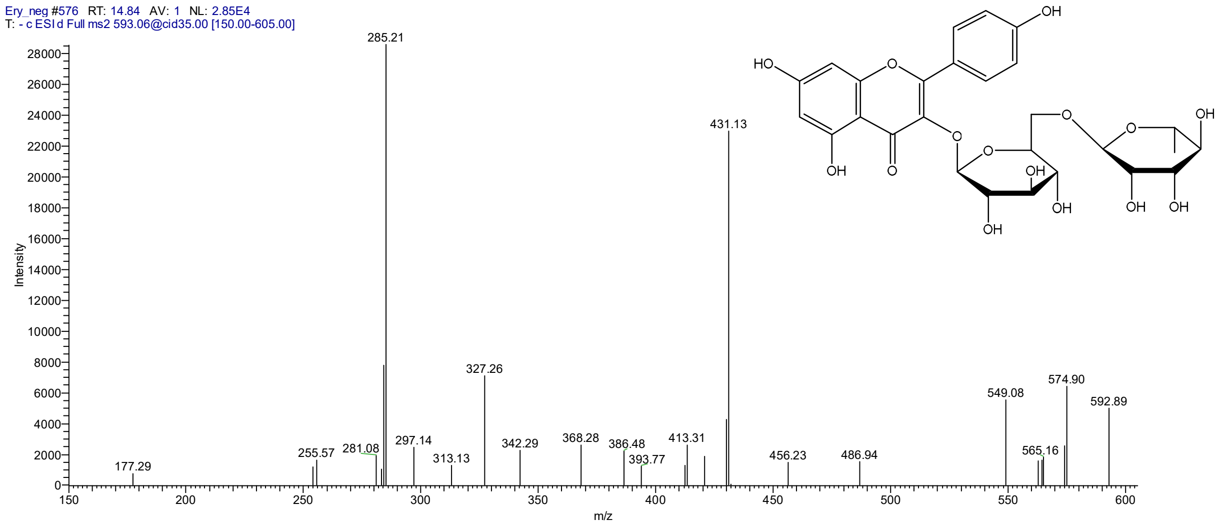

Figure S7. ESI-MS/MS spectrum of kaempferol-3-*O*-rutinoside (**18**) [M-H]$^-$ at *m/z* 593

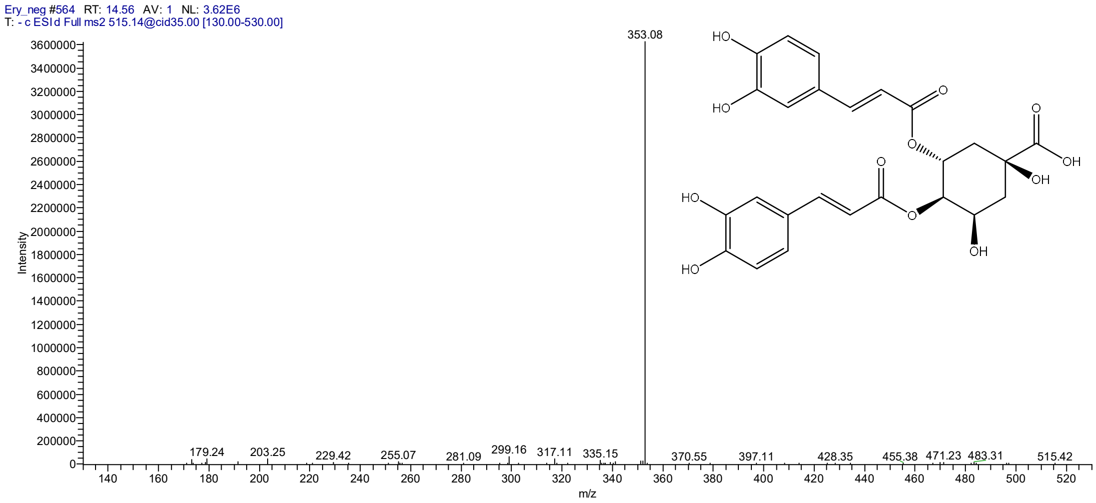

Figure S8. ESI-MS/MS spectrum of dicaffeoyl quinic acid (**19**) [M-H]$^-$ at *m/z* 515

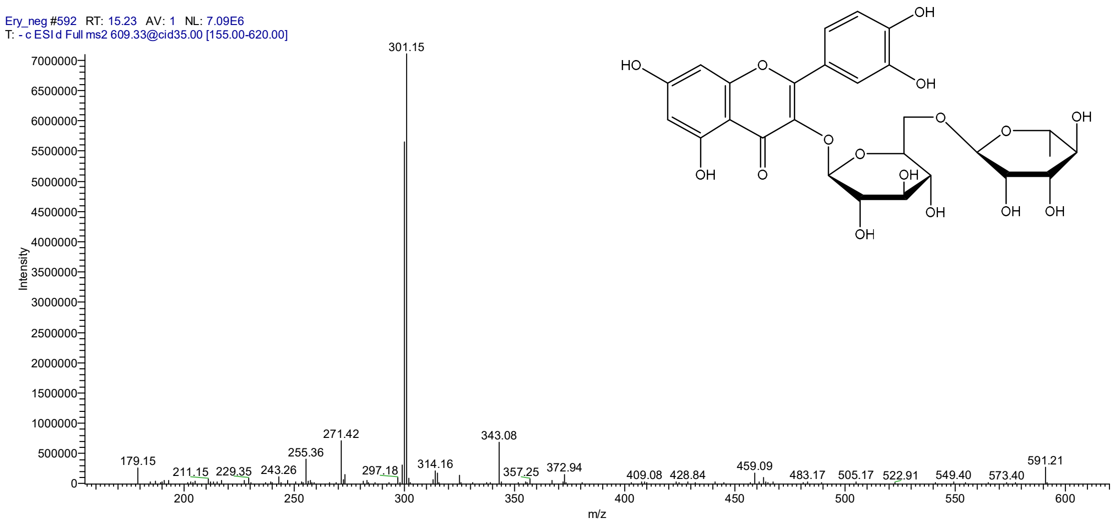

Figure S9. ESI-MS/MS spectrum of quercetin-3-*O*-rutinoside (**20**) $[M-H]^-$ at *m/z* 609

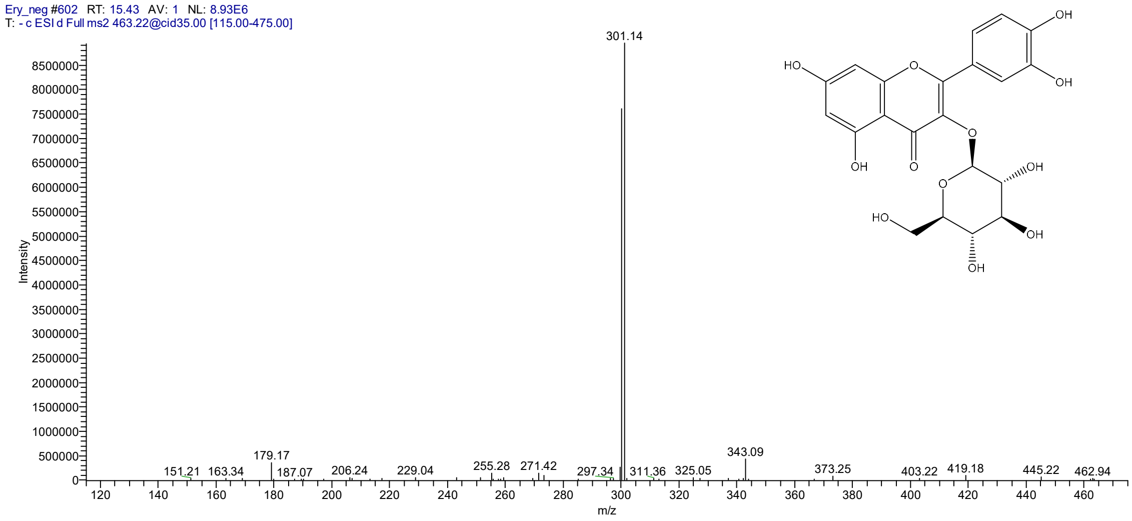

Figure S10. ESI-MS/MS spectrum of quercetin-3-*O*-hexoside (**21**) $[M-H]^-$ at *m/z* 463

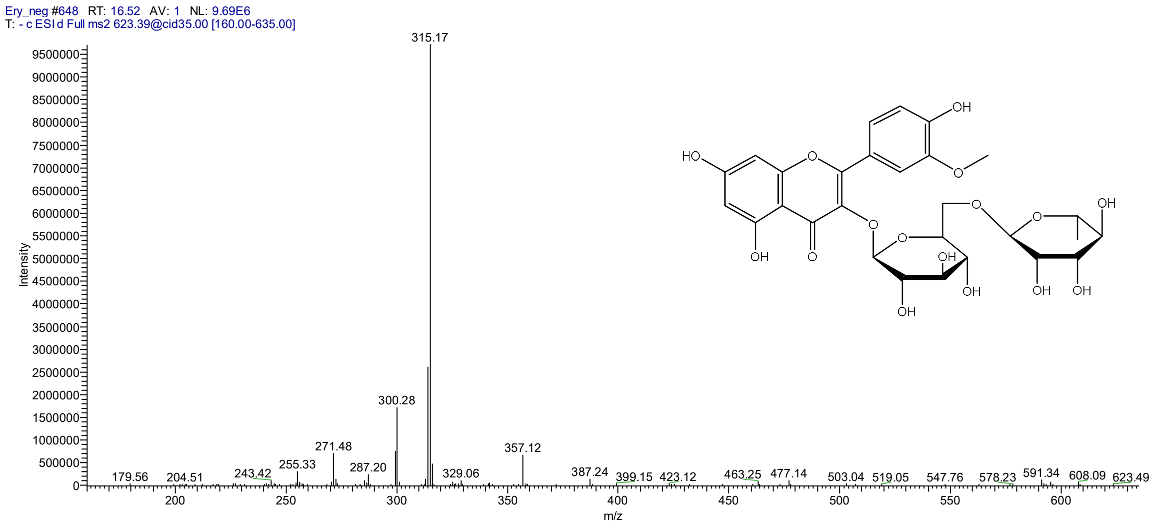

Figure S11. ESI-MS/MS spectrum of isorhamnetin-*O*-rutinoside (**22**) $[M-H]^-$ at *m/z* 623

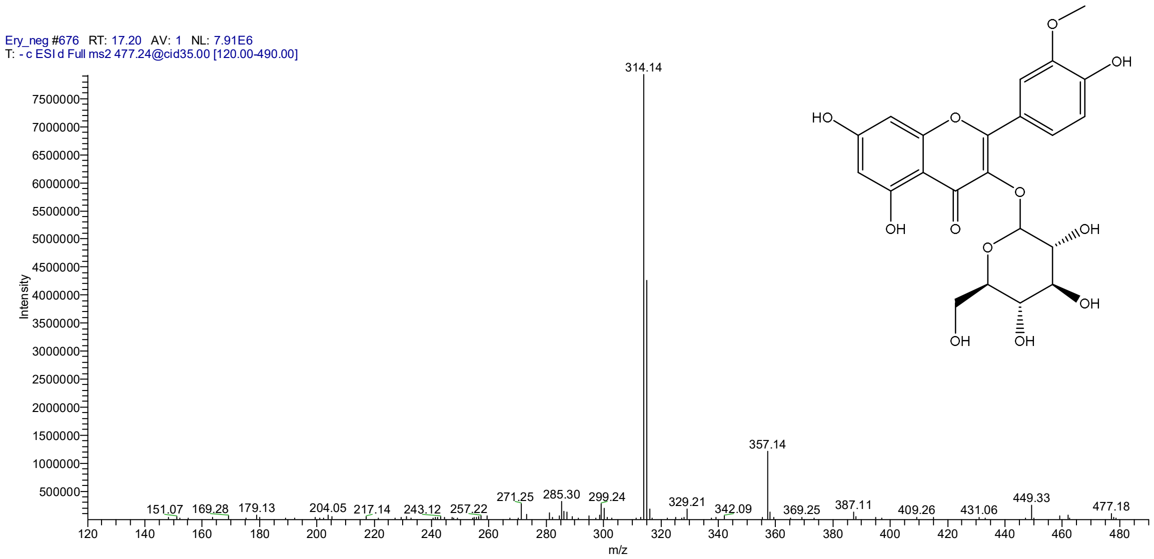

Figure S12. ESI-MS/MS spectrum of isorhamnetin-*O*-hexoside (**23**) $[M-H]^-$ at *m/z* 477
